# Atypical Gallstone Ileus in a Young Adult: A Diagnostic Challenge and Surgical Resolution

**DOI:** 10.7759/cureus.87928

**Published:** 2025-07-14

**Authors:** Haytham Mohammed Alzinati, Ahmed Zaki, Ahmed Fathi Mohamed Alsehily, Ahmed Elsayed Ibrahim Mattar, Ammar Mohamed Saleih Abdalah Basheir, Shahad Yaser Mustafa, Abdulmalek Ayman Arbach

**Affiliations:** 1 General Surgery, Saudi German Hospital Hail, Hail, SAU; 2 General Surgery, Sulaiman Al Rajhi University, Hail, SAU; 3 General Surgery, Ibn Sina University, Hail, SAU

**Keywords:** gall bladder disease, gall bladder diseases and gallstones, gall stone, intestinal obstruction, small intestinal obstruction

## Abstract

Gallstone ileus is a rare but serious complication of cholelithiasis resulting in mechanical bowel obstruction. We present the case of a 40-year-old woman with no prior medical history who developed classic features of gallstone ileus. Radiological investigations revealed Rigler’s triad (small bowel obstruction, pneumobilia, ectopic gallstone), and the patient underwent exploratory laparotomy and enterotomy with successful extraction of multiple gallstones. Postoperative recovery was uneventful, and this case emphasizes the importance of early imaging and surgical intervention in such cases.

## Introduction

Gallstone ileus is an uncommon but serious complication of cholelithiasis, described in detail in the surgical literature since the early 1990s [1]. Comprehensive reviews have since clarified its incidence and clinical features, noting that it represents <1%-4% of all mechanical bowel obstructions [2]. Its incidence rises to 14%-25% in elderly females with chronic gallbladder disease [1,3]. Subsequent studies and case reports have further explored the pathophysiology and management strategies, emphasizing the importance of early diagnosis and intervention [3,4]. The clinical presentation of gallstone ileus is often non-specific, mimicking other causes of bowel obstruction, which can lead to diagnostic delays [5,6]. Classic radiographic findings, such as pneumobilia and intestinal obstruction, are well-documented in the literature [7,8]. The advent of computed tomography (CT) has significantly improved diagnostic accuracy, making it the imaging modality of choice for this condition [9,10].

Management of gallstone ileus remains a subject of debate, with surgical options including enterotomy with stone extraction alone or combined procedures involving cholecystectomy and fistula closure [10,11]. The choice of surgical approach should be individualized, taking into account patient age, comorbidities, and the presence of residual stones or fistula. We present a case of gallstone ileus in an adult female without prior gallbladder disease, an exceptionally rare demographic for this condition. This report aims to highlight the diagnostic challenges, emphasize the importance of early intervention, and discuss the nuances of surgical management in atypical cases.

## Case presentation

A 40-year-old female with no significant past medical history presented to the emergency department with a 12-hour history of acute, severe colicky abdominal pain (initially epigastric, becoming diffuse), multiple episodes of bilious vomiting, and absolute constipation (no passage of flatus or stool). She reported no fever, jaundice, or prior similar episodes. On examination, she was alert, oriented, and anxious due to pain, with no signs of jaundice or dehydration. Vital signs were stable: blood pressure 130/70 mmHg, heart rate 80 beats per minute, respiratory rate 16/min, temperature 37.0°C, and oxygen saturation (SpO₂) 99% on room air. Abdominal examination revealed visible distension with diffuse tenderness, maximal in the lower quadrants. The abdomen was soft without guarding, rebound tenderness, or palpable masses. Bowel sounds were initially hyperactive but became diminished. Digital rectal examination revealed an empty rectum.

**Figure 1:**
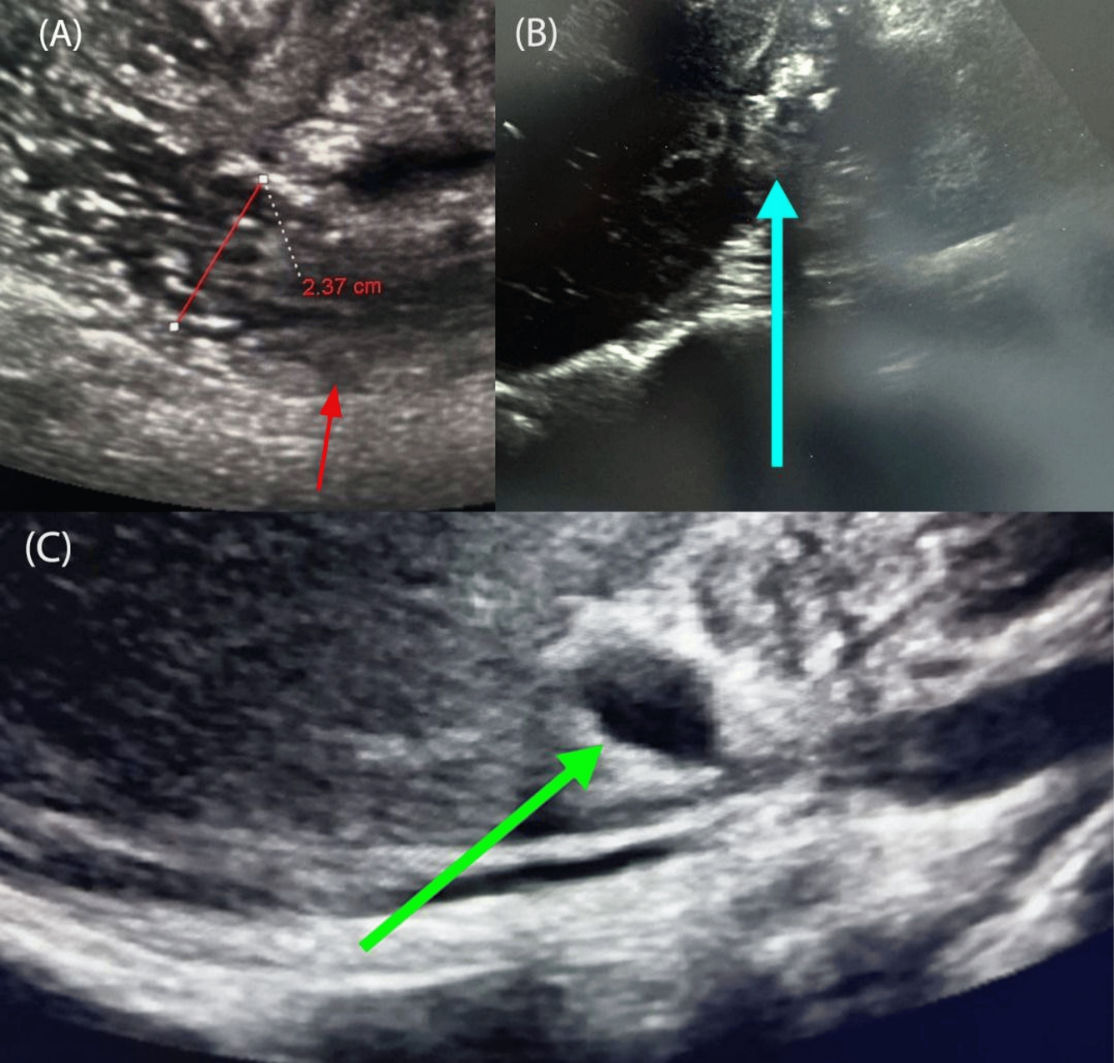
Abdominal ultrasound (A) showing dilated bowel loop (red arrow) and (B) contracted gallbladder with calculi (blue arrow), and (C) echogenic foci with "dirty" shadowing in the biliary tree (pneumobilia) identified by green arrow

**Figure 2:**
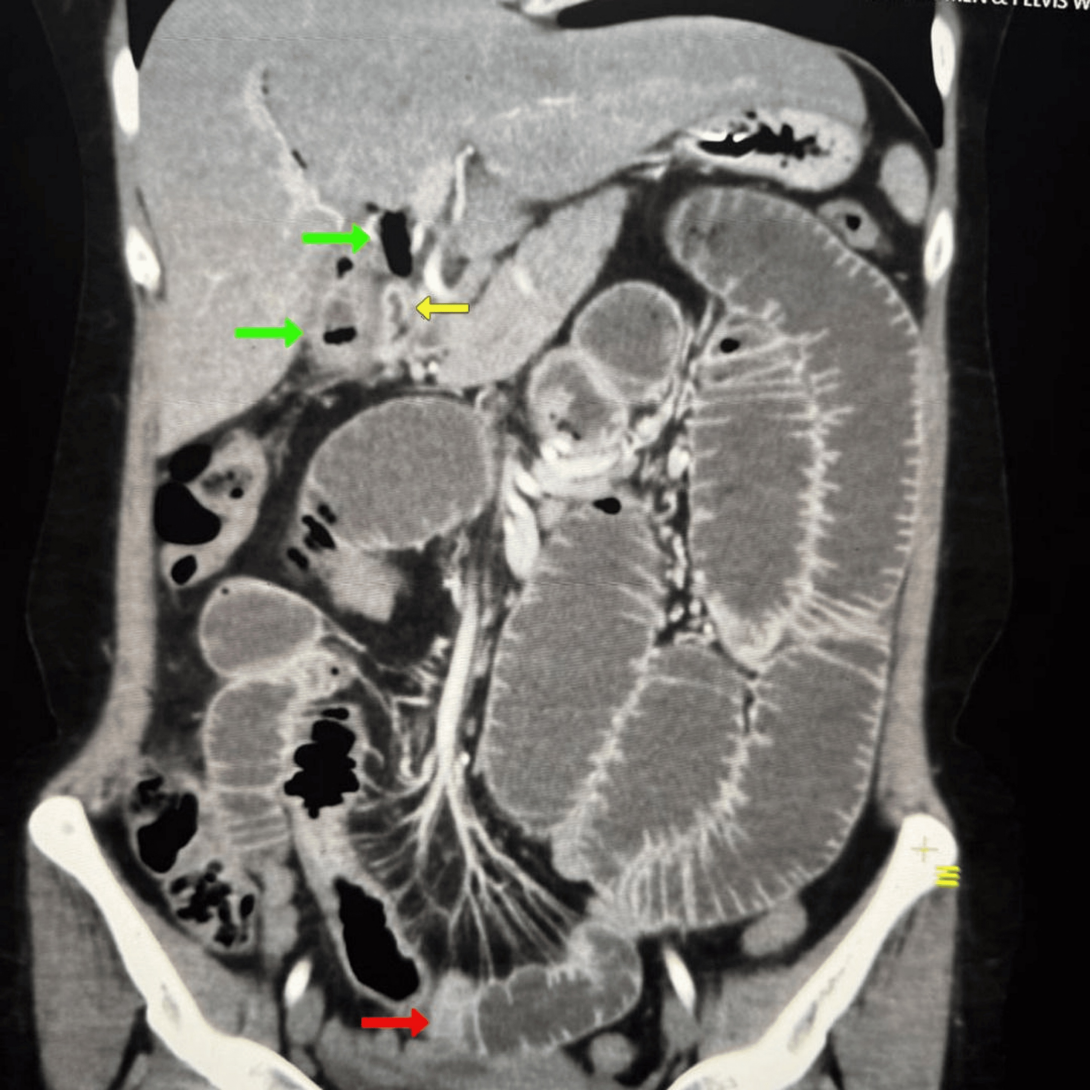
Contrast-enhanced CT demonstrating small bowel obstruction, pneumobilia (green arrow), and a 3x3 cm calcified intraluminal density (gallstone red arrow) in the distal ileum, along with cholecystoduodenal fistula (yellow arrow)

Initial laboratory investigations (Table 1) revealed leukocytosis (white blood cell count (WBC) 11.6 × 10³/μL, 67.8% neutrophils), elevated C-reactive protein (CRP) (48 mg/L), and mild hypokalemia (K⁻ 3.2 mmol/L). Abdominal ultrasound demonstrated dilated small bowel loops (>3 cm diameter) with sluggish peristalsis, a contracted gallbladder containing multiple shadowing calculi, and echogenic foci with "dirty" shadowing in the biliary tree suggestive of pneumobilia (Figures 1A-1C). Subsequent contrast-enhanced CT abdomen/pelvis confirmed small bowel obstruction with a transition point in the distal ileum, pneumobilia (Figure 2), and a 3x3 cm calcified intraluminal density impacted in the distal ileum approximately 60 cm proximal to the ileocecal valve (Figure 2). A cholecystoduodenal fistula was also visualized. These findings constituted Rigler's triad, confirming gallstone ileus.

**Table 1:** Laboratory investigations

Parameter	Result	Unit	Reference Range	Status
WBC (white blood cell count)	11.6	×10³/µL	4-11	High
RBC (red blood cell count)	6.01	×10³/µL	4.2-5.4	High
HGB (hemoglobin)	15.7	g/dL	12.5-16	Normal
HCT (hematocrit)	50	%	37-47	High
MCV (mean corpuscular volume)	83.2	fL	83-99	Normal
MCH (mean corpuscular hemoglobin)	26.1	pg	27-32	Low
MCHC (mean corpuscular hemoglobin concentration)	31.4	g/dL	31.5-34.5	Low
RDW (red cell distribution width)	13.2	%	11.5-14.5	Normal
PLT (platelet count)	323	×10³/µL	150-400	Normal
Neutrophils %	67.8	%	50-62	High
Lymphocytes %	19.1	%	25-40	Low
Monocytes %	10.7	%	3-7	High
Eosinophils %	0.65	%	0-6	Normal
Basophils %	1.72	%	0-1	High
Neutrophils (Abs, absolute count)	7.86	×10³/µL	2-7	High
Lymphocytes (Abs, absolute count)	2.22	×10³/µL	1.5-4	Normal
Monocytes (Abs, absolute count)	1.24	×10³/µL	0.1-0.8	High
Eosinophils (Abs, absolute count)	0.08	×10³/µL	0-0.4	Normal
Basophils (Abs, absolute count)	0.2	×10³/µL	0-0.2	Normal

**Figure 3:**
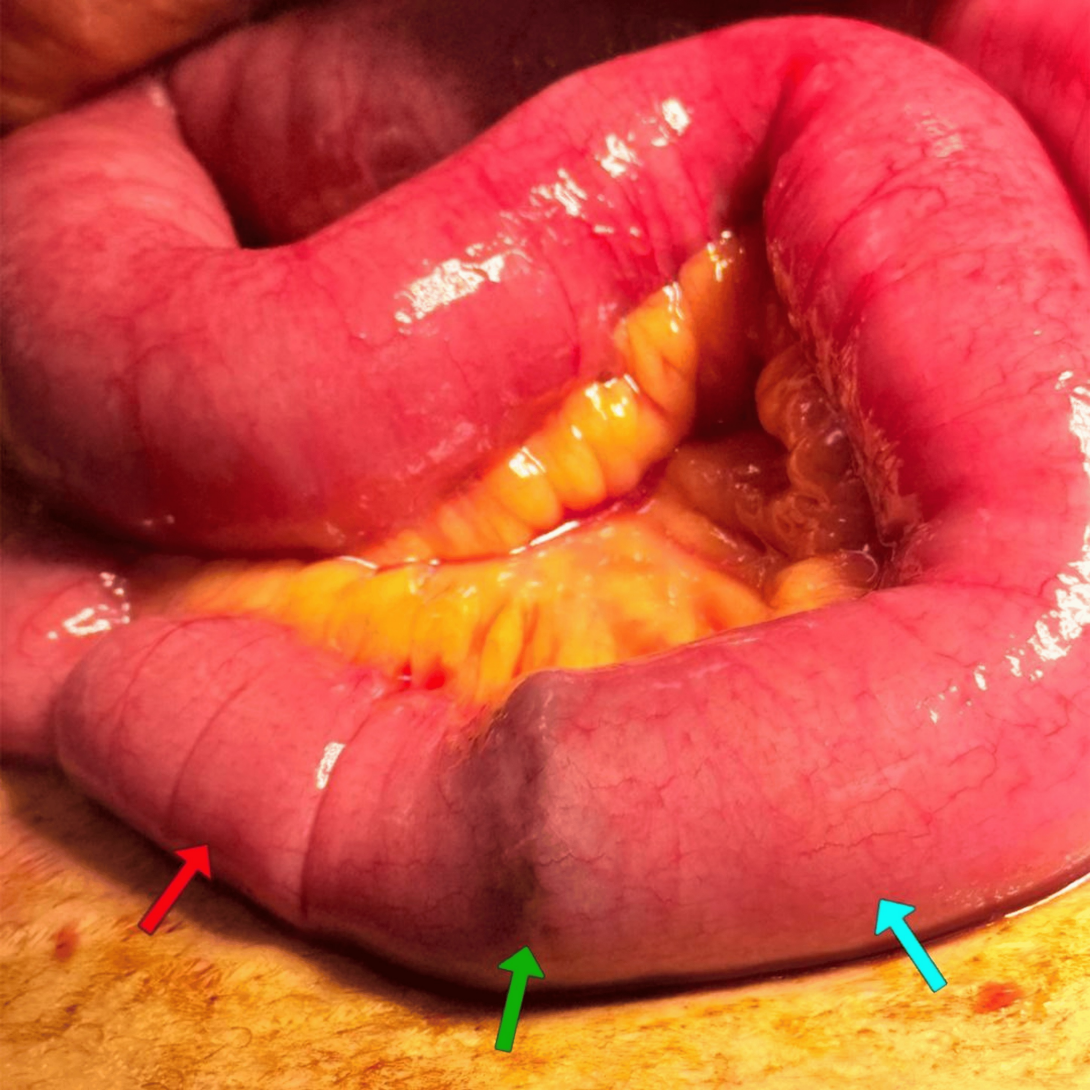
Intraoperative photograph illustrating dilated small bowel loops (blue arrow) proximal to the impacted stone (green arrow) in the distal ileum (~60 cm from ileocecal valve) and collapsed bowel distal (red arrow)

She was admitted to the intensive care unit (ICU) for preoperative optimization. Management included nil per os (NPO), intravenous fluid resuscitation, potassium supplementation, nasogastric tube decompression, intravenous antibiotics (Cefazolin 1 g every 8 h and Metronidazole 500 mg every 8 h), analgesia (intravenous (IV) Paracetamol and opioids), and deep vein thrombosis (DVT) prophylaxis (Enoxaparin 40 mg subcutaneously (SC) daily). On the same day , she underwent exploratory laparotomy. Operative findings included dilated small bowel loops proximal to an impacted stone in the distal ileum (~60 cm from ileocecal valve (ICV) (Figure 3) and a cholecystoduodenal fistula with a shrunken, fibrotic gallbladder. A longitudinal enterotomy 5 cm proximal to the stone allowed extraction of multiple gallstones (largest 3x3 cm). The enterotomy was closed primarily in two layers. The fistula was intentionally left in situ due to significant inflammation and adhesions. Two closed-suction drains were placed (pelvic cul-de-sac and Morrison's pouch).

Postoperatively, she remained hemodynamically stable. The nasogastric tube was removed on postoperative day 2 (POD 2) after return of bowel function. Diet was advanced from sips/clear liquids on postoperative day 2 (POD 2) to regular diet on postoperative day 5 (POD 5), which was tolerated well. Spontaneous bowel movement occurred on POD 3. Drains were removed sequentially, pelvic on postoperative day (POD 3) and Morrison's pouch on postoperative day 4 (POD 4) due to minimal output. Intravenous (IV) antibiotics were discontinued on postoperative 5 (POD 5) after completing a five-day course. Laboratory markers improved: WBC normalized to 8.2 x 10³/µL by postoperative day 3 (POD 3), and C-reactive protein (CRP) decreased to 6 mg/L by postoperative day 5 (POD 5). Analgesia was transitioned to oral agents (Paracetamol/nonsteroidal anti-inflammatory drugs (NSAIDs)), and Enoxaparin was continued.

She was discharged on postoperative day 6 (POD 6) in stable condition, tolerating a regular diet and ambulating independently with minimal incisional pain controlled by oral analgesia. Discharge plans included outpatient surgical follow-up in two weeks and discussion of elective laparoscopic cholecystectomy with fistula takedown at six to eight weeks post-discharge after resolution of inflammation.

## Discussion

Gallstone ileus is an uncommon but important cause of mechanical small bowel obstruction, accounting for 1%-4% of all mechanical obstructions, with a higher prevalence (up to 14%-25%) in elderly females and those with chronic gallbladder disease [1-3]. Gallstone ileus is seldom reported in young adults like our 40-year-old patient. This underscores a key clinical lesson: age should not limit the differential diagnosis when evaluating bowel obstruction.

The pathogenesis involves the formation of a biliary-enteric fistula, most commonly cholecystoduodenal, through which large gallstones migrate into the intestinal tract and ultimately cause obstruction, typically at the terminal ileum due to its narrow lumen [3,4]. In this case, computed tomography (CT) revealed Rigler’s triad (small bowel obstruction, pneumobilia, and ectopic gallstone), confirming the diagnosis [5,6]. While plain abdominal radiographs may demonstrate some features, computed tomography (CT) is now considered the gold standard for diagnosis, offering superior sensitivity and specificity for identifying all components of Rigler’s triad and delineating the anatomical relationships involved [7,8].

The clinical presentation of gallstone ileus is often non-specific, including intermittent abdominal pain, vomiting, and constipation, which may mimic other causes of bowel obstruction and contribute to diagnostic delays [4,5,9]. In the present case, early use of abdominal ultrasound followed by contrast-enhanced computed tomography (CT) allowed for timely diagnosis and expedited surgical intervention, which is critical for reducing morbidity and mortality.

Surgical management remains the cornerstone of treatment, but the optimal approach is debated. Options include simple enterolithotomy or a one-stage procedure combining enterolithotomy, cholecystectomy, and fistula closure [10,11]. Enterolithotomy alone is often favored in the acute setting, especially in patients with significant inflammation or comorbidities, as it is associated with shorter operative times and lower perioperative risk [3,10]. In this case, enterolithotomy with stone extraction and primary closure was performed, with the cholecystoduodenal fistula left in situ due to significant local inflammation. This approach is supported by current evidence, which suggests that delayed or elective management of the fistula may be considered once the acute process has resolved [9,11].

The patient's postoperative course was uncomplicated, with rapid normalization of laboratory markers and early return of bowel function. She was discharged on postoperative day 6 in stable condition, with plans for elective cholecystectomy and fistula repair after resolution of inflammation. This favorable outcome is consistent with recent literature supporting individualized, staged surgical management in selected cases [6,9].

This case highlights the importance of considering gallstone ileus in the differential diagnosis of small bowel obstruction, even in younger patients without evidence of prior biliary disease. Early imaging, multidisciplinary management, and tailored surgical intervention are essential for optimizing outcomes. The increasing use of advanced imaging modalities such as computed tomography (CT) has significantly improved diagnostic accuracy and facilitated timely intervention [1,3,7,10].

## Conclusions

Gallstone ileus, while rare and typically seen in elderly patients, can present in younger adults and should be considered in cases of unexplained small bowel obstruction. Early recognition and prompt surgical intervention are crucial to improving patient outcomes. Advanced imaging, particularly computed tomography (CT), plays a pivotal role in diagnosis. Individualized surgical management, often favoring enterolithotomy alone in the acute setting, can lead to excellent recovery, with elective management of the biliary fistula considered after resolution of the acute process. This case reinforces the need for high clinical suspicion and a multidisciplinary approach in atypical presentations of gallstone ileus.
